# Supplementary figures and images for: A resistome survey across hundreds of freshwater bacterial communities reveals the impacts of veterinary and human antibiotics use
Source: Front Microbiol. 2022 Oct 6;13:995418. doi: 10.3389/fmicb.2022.995418 (PMC9629221; doi:10.3389/fmicb.2022.995418)

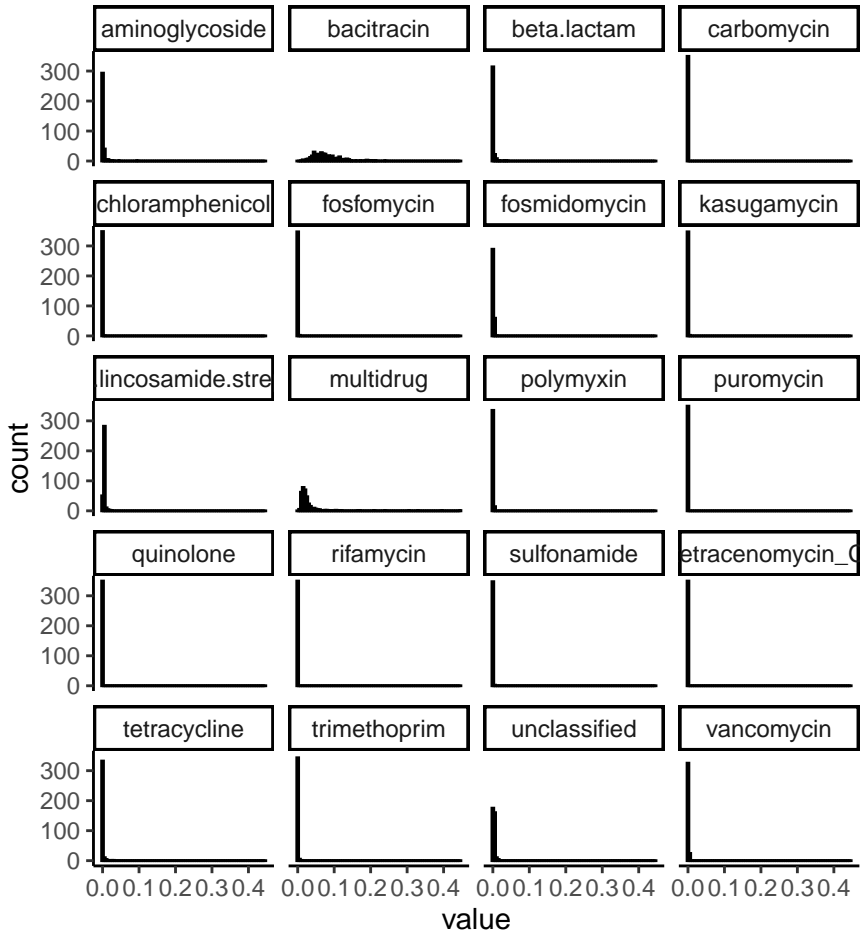

Supplement: Supplementary file 5 [file Data_Sheet_1.PDF]
